# Maternal proviral load and vertical transmission of Human T cell Lymphotropic Virus type 1 in Guinea-Bissau

**DOI:** 10.1186/1742-4690-8-S1-A72

**Published:** 2011-06-06

**Authors:** Carla van Tienen, Samuel J McConkey, Thushan I de Silva, Matthew Cotten, Steve Kaye, Ramu Sarge-Njie, Carlos da Costa, Nato Gonçalves, Julia Parker, Tim Vincent, Assan Jaye, Peter Aaby, Hilton Whittle, Maarten Schim van der Loeff

**Affiliations:** 1 Viral diseases program, Medical Research Council, Fajara, Gambia; 2 Department of Medical Microbiology and Infectious Diseases, Erasmus Medical Centre, Rotterdam, Netherlands; 3 Centre for Medical Molecular Virology, Division of Infection and Immunity, University College London, London, UK; 4 Projecto de Saúde de Bandim, Bissau. Guinea-Bissau; 5 Projecto de Saúde de Bandim/Indepth Network, Bissau, Guinea-Bissau; 6 Municipal Health Services, Amsterdam, Netherlands

## Background

The relative importance of routes of transmission of Human T cell Lymphotropic Virus type 1 (HTLV-1) in Guinea-Bissau is largely unknown; vertical transmission is thought to be important, but there are very few existing data. We aimed to examine factors associated with transmission in mothers and children in Guinea-Bissau, where HTLV-1 is endemic (prevalence of 5%).

## Methods

A cross-sectional survey was performed among mothers and their children (aged <15 years) in a rural community in Guinea-Bissau. A questionnaire to identify risk factors for infection and a blood sample were obtained. HTLV-1 proviral load in peripheral blood was determined and PCR was performed to compare Long Terminal Repeat (LTR) sequences in mother-child pairs.

## Results

Fourteen out of 55 children (25%) of 31 HTLV-1 infected mothers were infected versus none of 70 children of 30 uninfected mothers. The only factor significantly associated with HTLV-1 infection in the child was the proviral load of the mother; the risk of infection increased significantly with the log10 proviral load in the mother's peripheral blood (OR 5.5, 95% CI 2.1-14.6, per quartile), adjusted for weaning age and maternal income. HTLV-1 sequences of the LTR region obtained from mother-child pairs were identical within pairs but differed between the pairs.

## Conclusions

Vertical transmission plays an important role in HTLV-1 transmission in this community in Guinea-Bissau. The risk of transmission increases with the mother’s proviral load in the peripheral blood. Identical sequences in mother-child pairs give additional support to the maternal source of the children's infection.

